# Genome-wide identification of PDX and expression analysis under waterlogging stress exhibit stronger waterlogging tolerance in transgenic *Brassica napus* plants overexpressing the *BnaPDX1.3* gene compared to wild-type plants

**DOI:** 10.3389/fpls.2025.1533219

**Published:** 2025-02-12

**Authors:** Mingyao Yao, Bo Hong, Hongfei Ji, Chunyun Guan, Mei Guan

**Affiliations:** ^1^ College of Agriculture, Hunan Agricultural University, Changsha, China; ^2^ Hunan Branch of National Oilseed Crops Improvement Center, Changsha, China; ^3^ Southern Regional Collaborative Innovation Center for Grain and Oil Crops in China, Changsha, China

**Keywords:** Brassica napus, BnaPDX1.3, waterlogging stress, vitamin B6, overexpression

## Abstract

The *PDX* gene is a key gene in the vitamin B6 synthesis pathway, playing a crucial role in plant growth, development, and stress tolerance. To explore the family characteristics of the *PDX* gene in *Brassica napus* (*B. napus*) and its regulatory function under waterlogging stress, this study used five *PDX* genes from Arabidopsis thaliana as the basis for sequence analysis. Thirteen, eight, and six *PDX* genes were identified in *B. napus*, *Brassica oleracea* (*B. oleracea*), and *Brassica rapa* (*B. rapa*), respectively. Bioinformatics study reveals high conservation of *PDX* subfamily genes during evolution, and *PDX* genes in *B. napus* respond to waterlogging stress.In order to further investigate the effect of the *PDX* gene on waterlogging tolerance in *B. napus*, expression analysis was conducted on *BnaPDX1.3* gene overexpressing *B. napus* plants and wild-type plants. The study showed that overexpressing plants could synthesize more VB6 under waterlogging stress, exhibit stronger antioxidant enzyme activity, and have a more effective and stable ROS scavenging system, thus exhibiting a healthier phenotype. These findings suggested that the *BnaPDX1.3* gene can enhance the waterlogging tolerance of *B. napus*, which is of great significance for its response to waterlogging stress. Our study provides a basic reference for further research on the regulation mechanism of the *PDX* gene and waterlogging tolerance in *B. napus*.

## 1 Introduction

Vitamin B6 is an essential water-soluble vitamin required by all living organisms (Gorelova et al., 2022). It is recognized as an antioxidant and is linked to responses to various biotic and abiotic stresses (Vanderschuren et al., 2013; Samsatly et al., 2016; Chung, 2012; Zhang et al., 2015; Asensi-Fabado and Munné-Bosch, 2010). Vitamin B6 consists of six interconvertible compounds: pyridoxine phosphate (PNP), pyridoxal phosphate (PLP), pyridoxamine phosphate (PMP), and their nonphosphorylated derivatives (pyridoxine [PN], pyridoxal [PL], and pyridoxamine [PM]) (Fitzpatrick et al., 2007; Roje, 2007; Mooney et al., 2009). Additionally, vitamin B6 must be phosphorylated to function as a coenzyme (Colinas et al., 2016). Among them, PLP is the most important coenzyme, playing a crucial role in lipid degradation and carbohydrate storage (such as glycogen) (Jeong and VacantiLi, 2020; Mooney et al., 2009; Rueschhoff et al., 2013). It also plays a decisive role in amino acid metabolism, catalyzing transamination, decarboxylation, and α,β-elimination reactions involved in amino acid metabolism (Eliot and Kirsch, 2004; Drewke and Leistner, 2001). Other studies have found that the five enzymes that most commonly use PLP as a coenzyme, in order of dependency, are transferases, lyases, isomerases, hydrolases, and oxidoreductases, demonstrating the versatility of PLP-dependent enzymes (Percudani and Peracchi, 2003).

In plants, the *de novo* biosynthesis pathway of vitamin B6 relies on two proteins, *PDX1* and *PDX2*, which function as glutaminyltransferases (Tambasco-Studart et al., 2005, 2007). *PDX2* possesses transaminase activity, extracting ammonium groups from glutamine and incorporating them into the product. *PDX1* receives these ammonium groups and synthesizes the final product (Raschle et al., 2005; Dong et al., 2004). In the *AtPDX* family of *Arabidopsis thaliana*, there are three *AtPDX1* (*AtPDX1.1*, *AtPDX1.2*, and *AtPDX1.3*) and one *AtPDX2* protein member (Tambasco-Studart et al., 2005; Wetzel et al., 2004). However, only *PDX1.1* and *PDX1.3* are involved in the biosynthesis of vitamin B6, while *PDX1.2* does not play a role in this process (Tambasco-Studart et al., 2005; Titiz et al., 2006). Instead, *PDX1.2* functions as a pseudoenzyme that enhances the activity of catalytic homologs under stress conditions (Moccand et al., 2014). Studies on *pdx1* and *pdx2* mutants in *Arabidopsis thaliana* have shown that knocking out both genes, *PDX1.1* and *PDX1.3*, or knocking out the single gene, *PDX2*, leads to the death of the mutant at the embryonic stage of development (Tambasco-Studart et al., 2005; Titiz et al., 2006). However, single mutants of *pdx1.1* or *pdx1.3* can survive, although with a short root phenotype, with the latter phenotype being more pronounced (Titiz et al., 2006; Chen and Xiong, 2005; Wagner et al., 2006; Boycheva et al., 2015). Hao Chen et al. (2009) suggested that the short-root phenotype in *pdx1* mutants was caused by reduced levels of endogenous auxin synthesis (Chen and Xiong, 2009). In *Arabidopsis thaliana*, the expression level of *PDX1.3* is consistently higher than that of *PDX1.1*, both spatially and temporally. Additionally, *pdx1.3* mutants exhibit more pronounced phenotypic differences in morphology and development compared to *pdx1.1* mutants (Titiz et al., 2006), despite the two proteins being 87% identical and capable of synthesizing vitamin B6 at comparable rates (Tambasco-Studart et al., 2005). In addition, Leuendorf et al. (2014) demonstrated that the lack of *PDX1.2* affects seed and hypocotyl development and results in a large number of aborted seeds during embryonic development, as shown through T-DNA insertion-generated heterozygous *PDX1.2* and artificial microRNA-reduced *PDX1.2* expression.

The *PDX* gene is not only crucial for the growth and development of plants but also indispensable for their response to adverse environmental stresses. Ristilä et al. (2011) discovered that *PDX1.3* is involved in plants’ response to UV-B radiation, with the formation of UV-B-induced *PDX1.3* primarily occurring in the parts of leaves that absorb UV-B radiation (Ristilä et al., 2011). It has also been proven that the *pdx1.3* mutant of *Arabidopsis thaliana* exhibits weaker tolerance to strong light and photo-oxidation (Havaux et al., 2009). Additionally, the *PDX1.2* gene of *Arabidopsis thaliana* has been found to exhibit higher expression under UV-B treatment, oxidative stress, and heat shock (Denslow et al., 2007). Furthermore, Dell’Aglio et al. (2017) identified a heat stress transcription initiation site within *PDX1.2*, and subsequent research indicated that *PDX1.2* can stably catalyze *PDX1* homologs under heat stress conditions.

Research on vitamin B6 (VB6) and *PDX* genes primarily focuses on their roles in plant growth, development, and stress resistance. However, limited studies have examined their roles in the response of *Brassica napus* (*B. napus*) to waterlogging stress. In this study, we investigated the impact of *BnaPDX1.3* gene overexpression (PDX1.3#20 and PDX1.3#21) and WT *B. napus* plants on waterlogging tolerance.

## 2 Materials and methods

### 2.1 Experimental materials

The *B. napus* variety G218 was provided by the Hunan Branch of the National Oil Crop Improvement Center.

### 2.2 *PDX* gene family analysis

Information related to the *PDX* gene of *Arabidopsis thaliana*, *Brassica rapa* (*B. rapa*), *Brassica oleracea* (*B. oleracea*), and *B. napus* was obtained from the Ensembl Plants database (http://plants.ensembl.org/index.html), including the full-length DNA sequence, CDS sequence, amino acid sequence, etc. The nucleic acid sequence and amino acid sequences of the *PDX* gene of *Arabidopsis thaliana* were first downloaded from Ensembl Plants. BLASTP alignment was performed on the genomes of *B. rapa*, *B. oleracea*, and *B. napus* with a threshold of *E* < 10^−5^ to identify *PDX* homologous genes. A phylogenetic tree was then constructed using the Neighbor-Joining (NJ) method in MEGA 11 (Tamura et al., 2021). Subsequently, Evolview (http://www.evolgenius.info/evolview/) was used to enhance the visualization of the phylogenetic tree (Subramanian et al., 2019). Motif analysis was conducted on the *PDX* gene of *Arabidopsis thaliana*, *B. rapa*, *B. oleracea*, and *B. napus* using MEME (http://MEME.nbcr.net/MEME/cgi-bin/MEME.cgi) (Bailey et al., 2015). Chromosome position information for the *PDX* gene was obtained from the gtf file of the Ensembl Plants database, and chromosome localization was analyzed using TBtools software (Chen et al., 2020). *Cis*-element analysis was conducted on the promoter region of the DNA sequence upstream 2,000 bp of the *PDX* gene of *Arabidopsis thaliana*, *B. rapa*, *B. oleracea*, and *B. napus* using PlantCARE (http://bioinformatics.psb.ugent.be/webtools/plantcare/html/) (Lescot et al., 2002). The expression pattern of the *BnaPDX* gene under waterlogging stress was analyzed based on the data from Hong et al. (2023). The results of gene structure, conserved domains, chromosome localization, *cis*-elements in the promoter region, and RNA-seq analysis were displayed using TBtools software (Chen et al., 2020).

### 2.3 qRT-PCR measurement

Total RNA was extracted from *B. napus* tissues using an assay kit from Vazyme Biotech Co. Ltd. (Nanjing, China) and reverse transcribed into cDNA with a reverse transcription assay kit from Takara Biomedical Technology (Beijing, China) Co. Ltd. Based on the reference gene sequence of *BnaPDX1.3* in *B. napus*, specific fluorescent quantitative primers were designed using the Primer-BLAST tool on the NCBI website (**Supplementary Table S1**). Following the instructions of the fluorescent quantitative PCR assay kit from TransGen Biotech Co. Ltd. (Beijing China)., the gene expression was detected using a Bio-Rad fluorescent quantitative PCR instrument. The data were analyzed using the 2^−ΔΔT^ method, with the *Bnactin* gene as the internal reference gene, to determine the relative expression level of the gene (**Supplementary Table S1**) (Livak and Schmittgen, 2001).

### 2.4 Construction of overexpression vectors and *Brassica napus* transformation

Based on the PC2300S vector map, we selected the *Kpn*I and *Xba*I restriction sites for constructing the *35S::BnaC03.PDX1.3* overexpression vector. We amplified the target fragment containing the restriction sites (**Supplementary Table S2**; **Supplementary Figure S1**), ligated the target vector with T4 ligase after restriction digestion, and used double enzyme digestion to verify the construction status of the vector (**Supplementary Figure S2**). We selected the successfully constructed recombinant overexpression vector, transferred it into *B. napus* G218 via *Agrobacterium*-mediated transformation, and performed positive detection on the transformed seedlings using specific primers for the *NptII* gene (**Supplementary Table S3**; **Supplementary Figure S3**). We used designed specific primers for the *35S::BnaC03.PDX1.3* recombinant overexpression vector to perform positive detection on T1 transgenic plants (**Supplementary Table S3**; **Supplementary Figures S4, S5**) and screened for *BnaPDX1.3* gene overexpression in the T1 generation plants.

### 2.5 Biomass measurement

Two groups of T1 generation *B. napus* G218 plants overexpressing the *BnaPDX1.3* gene and one group of G218 wild-type plants were subjected to 15 days of waterlogging stress. After the treatment, the fresh weight of the plants from all three groups was measured, and biomass analysis was conducted.

### 2.6 Vitamin B6 content measurement

Leaf tissues were collected from two groups of *BnaPDX1.3*-overexpressing *B. napus* plants and one group of wild-type plants after exposure to waterlogging for 0, 2, 4, 6, 9, 12, and 15 days. These tissues were flash-frozen in liquid nitrogen and stored at − 80°C. The VB6 content of the samples was detected using a vitamin B6 assay kit provided by ZCIBIO Technology Co. Ltd. (Shanghai, China).

### 2.7 Antioxidant enzyme activity and H_2_O_2_ content measurement

Leaf tissues were collected from two groups of *BnaPDX1.3*-overexpressing *B. napus* plants and one group of wild-type plants after exposure to waterlogging for 0, 2, 4, 6, 9, 12, and 15 days, respectively. The tissues were flash-frozen in liquid nitrogen and stored at − 80°C. The antioxidant enzyme activity and H_2_O_2_ content of the samples were detected using the SOD, POD, CAT, and H_2_O_2_ assay kits provided by ZCIBIO Technology Co. Ltd.

## 3 Statistical analysis

All measurements were conducted using three biological replicates. One-way ANOVA followed by Dunnett’s multiple comparisons test was performed using GraphPad Prism (v. 25), with *p* < 0.05 considered significant for all experiments.

## 4 Results

### 4.1 *PDX* gene family analysis

#### 4.1.1 Phylogenetic analysis of *PDX* proteins in *B. napus*, *B. rapa*, and *B. oleracea*

**Figure 1:**
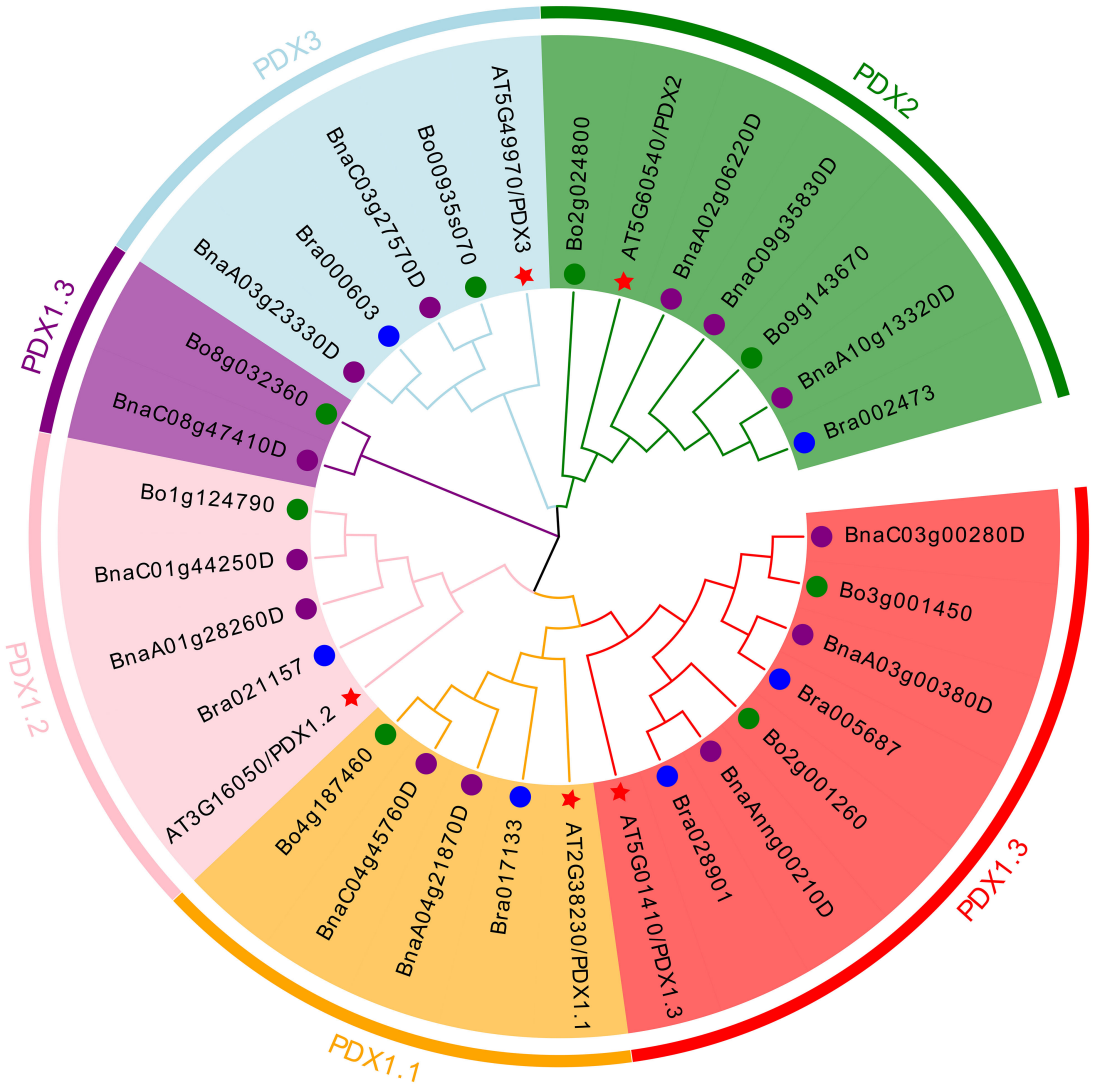
Phylogenetic tree of *PDX* proteins from *B. napus*, *B. rapa*, *B. oleracea*, and *Arabidopsis*. The neighbor-joining phylogenetic tree shows the relationships among 13 *B. napus*, six *B. rapa*, eight *B. oleracea*, and five *Arabidopsis PDX* proteins.

Conducting a BLAST search on the protein sequences (*ATPDX*) of five *Arabidopsis thaliana PDX* genes yielded six *B. rapa* genes (*BraPDX*), eight *B. oleracea* genes (*BoPDX*), and 13 *B. napus* genes (*BnaPDX*), all obtained from the Ensembl Plants database. Based on the *PDX* protein sequences from the four species, an evolutionary tree (**Figure 1**) was constructed using MEGA 11 software and the Evolview online beautification tool, visually reflecting the evolution and classification of the 32 *PDX* family members. According to the evolutionary tree analysis, the *PDX* proteins were divided into five subclades (*PDX1.1*, *PDX1.2*, *PDX1.3*, *PDX2*, *PDX3*), with two branches in *PDX1.3*. All five subclades contained genes from *B. rapa*, *B. oleracea*, and *B. napus*.

#### 4.1.2 Motif analysis (MEME) and gene structural analysis of *PDX*

**Figure 2:**
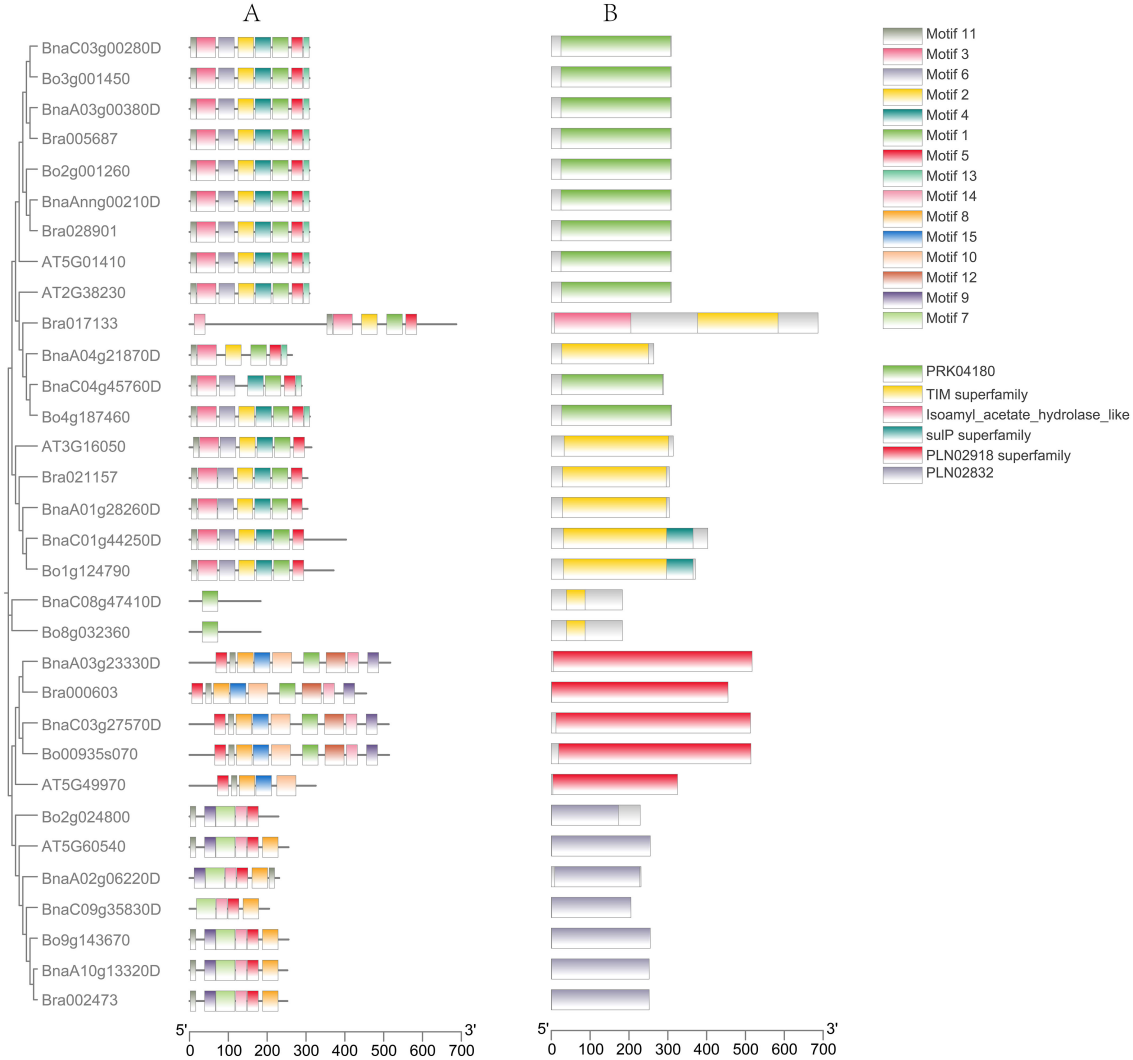
Gene motifs **(A)** and gene structure **(B)** analysis of *PDX* proteins. Fifteen motifs in *PDX* proteins were identified using MEME tools **(A)**. Orange boxes, black lines, and green boxes indicate exons, introns, and untranslated regions, respectively **(B)**.

To further investigate the evolutionary relationships among the *PDX* gene families of *Arabidopsis thaliana*, *B. rapa*, *B. oleracea*, and *B. napus*, we conducted a predictive analysis of their gene structures and motifs. Using MEME, we analyzed 15 motifs among 32 proteins (**Figure 2**). It was found that members belonging to the same subfamily exhibited certain similarities in their conserved motifs. The conserved motifs of most *PDX1.1* and *PDX1.3* proteins were arranged as Motif11-Motif3-Motif6-Motif2-Motif4-Motif1-Motif5-Motif13, with the exception of *Bra017133*, which lacked Motif4, Motif6, and Motif13; *BnaA04g21870D*, which lacked Motif4 and Motif6; *BnaC04g45760D*, which lacked Motif2; and *BnaC08g47410D* and *Bo8g032360*, which retained only the core conserved motif, Motif1, of the pyridoxal phosphate synthase domain. This may also imply that they still perform functions related to vitamin B6 synthesis. Compared to *PDX1.1* and *PDX1.3*, the conserved motif of the *PDX1.2* protein lacked Motif13, with its composition pattern being Motif11-Motif3-Motif6-Motif2-Motif4-Motif1-Motif5. In summary, the functions of *PDX1.1*, *PDX1.2*, and *PDX1.3* proteins should be similar.

Except for *AT5G49970*, which lacks Motif1, Motif9, Motif12, and Motif14, the conserved motif patterns of the remaining *PDX2* proteins are Motif5-Motif11-Motif8-Motif15-Motif10-Motif1-Motif12-Motif14-Motif9. Most *PDX3* proteins have conserved motif patterns of Motif11-Motif9-Motif7-Motif14-Motif5-Motif8, with *Bo2g024800* lacking Motif8 and *BnaC09g35830D* lacking Motif9 and Motif11. The motif prediction results are generally consistent with the evolutionary tree alignment analysis, suggesting that *PDX1* genes from different subclades may perform similar functions while exhibiting significant differences from the other two major clades. This motif specificity distribution among different subclades may reflect the functional differentiation of *BnaPDX* genes in *B. napus*.

Gene structure prediction reveals that the structure of the *PDX1* genes (*PDX1.1*, *PDX1.2*, *PDX1.3*) is relatively conserved, with most introns ranging from 0 to 2 in number. Specifically, 13 *PDX1* genes lack introns, three *PDX1* genes possess one intron and two exons, three *PDX1* genes have two introns and three exons, and only *Bra017133* features nine introns and 10 exons. Meanwhile, the gene structure of the *PDX2* subgroup remains consistent, exhibiting a structure with 12 introns and 13 exons. Within the *PDX3* subgroup, two types exhibit four introns and five exons: *Bo2g024800* and *BnaA02g06220D*, while the rest possess a structure with five introns and six exons.

#### 4.1.3 Chromosomal distribution of *PDX* genes

**Figure 3:**
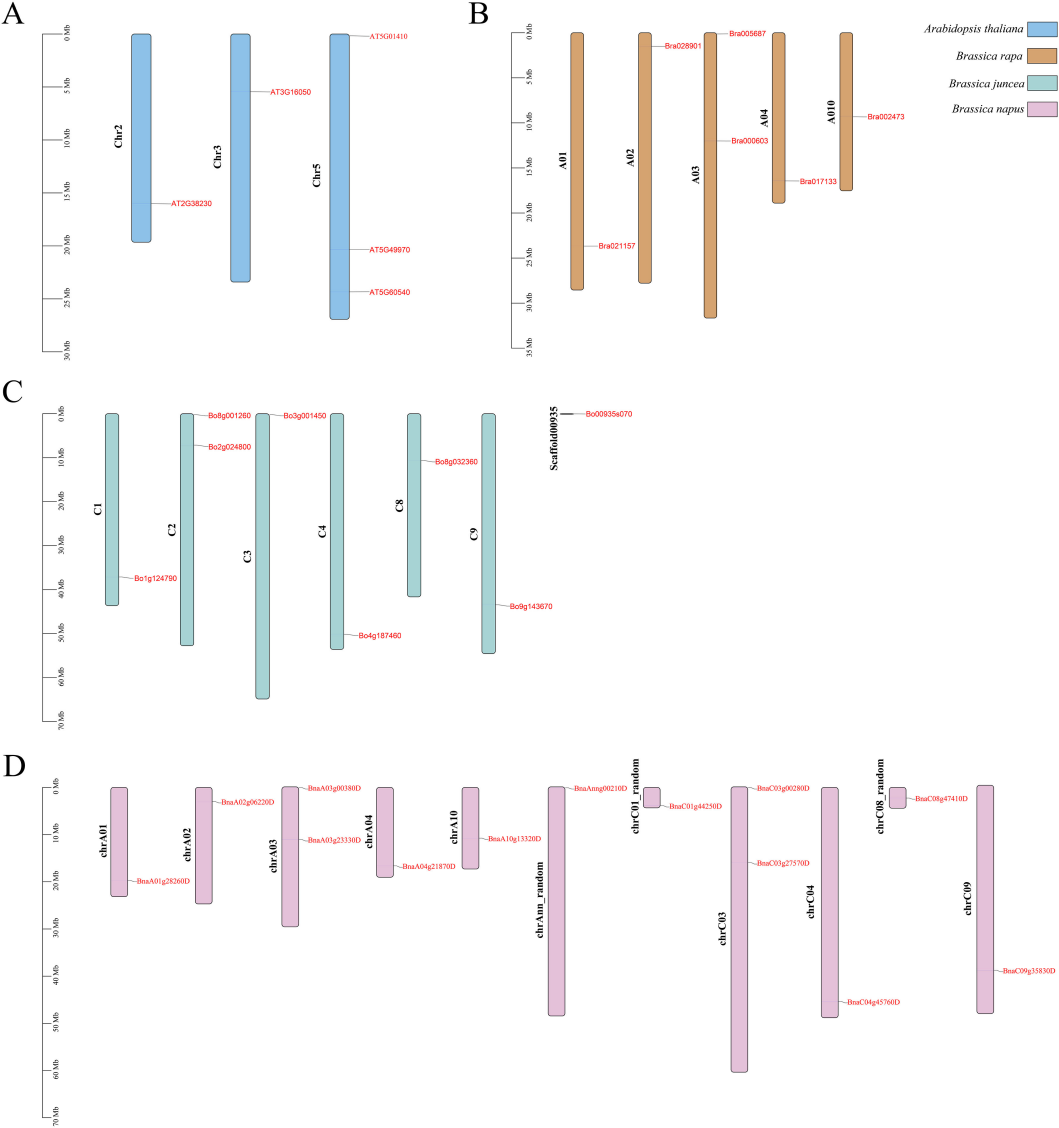
Chromosomal distribution of *PDX* genes in *Arabidopsis* **(A)**, *B. rapa* **(B)**, *B. oleracea* **(C)**, and *B. napus* **(D)**.

The *PDX* gene does not exhibit tandem repeats in the four species. Five *Arabidopsis thaliana ATPDX* genes are located on chromosomes Chr2, Chr3, and Chr5; six Chinese *B. rapa BraPDX* genes are located on chromosomes A01, A02, A03, A04, and A10; and eight *B. oleracea BoPDX* genes and 13 *B. napus BnaPDX* genes are located on seven and 11 chromosomes, respectively (**Figure 3**).

#### 4.1.4 The *cis*-element regulators in *BnaPDX1.3* promoters

**Figure 4:**
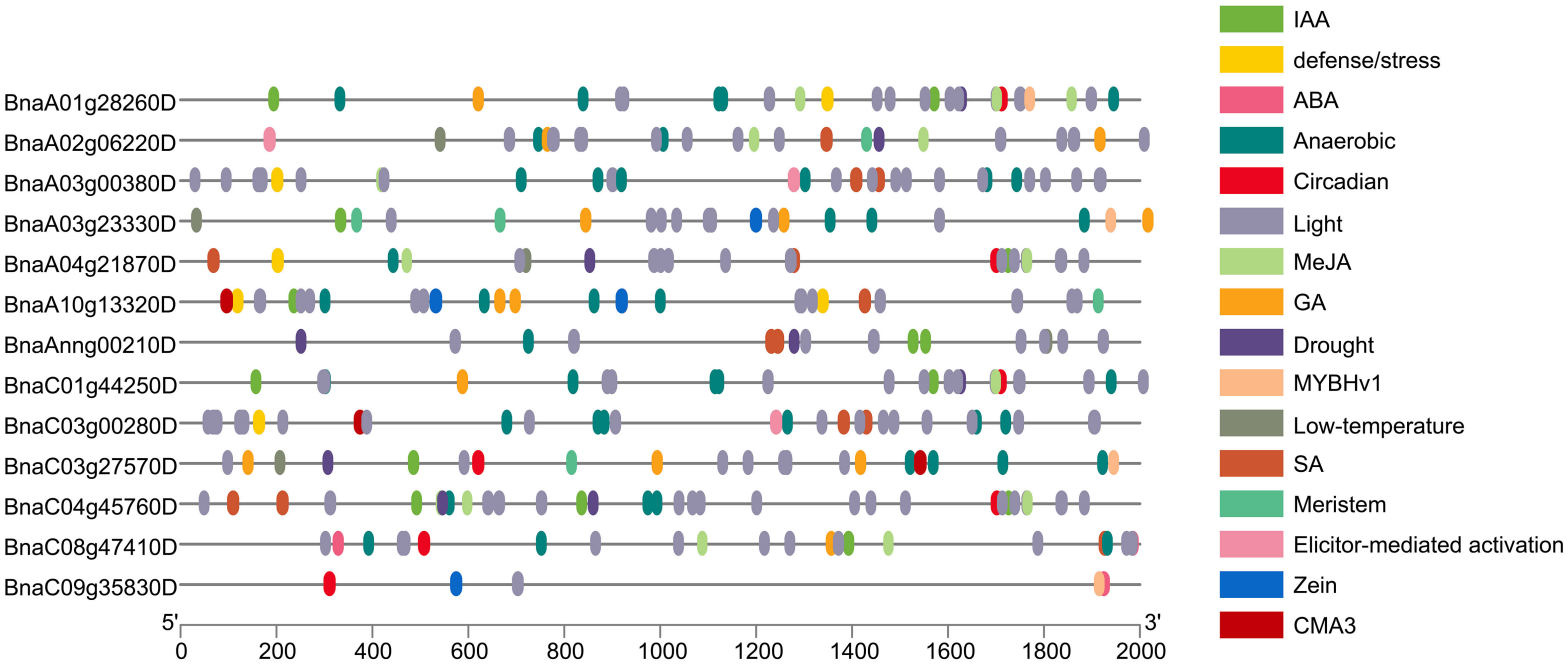
Promoter *cis*-element analysis of *PDX* genes in *B. napus*. Differently colored boxes represent various *cis*-acting elements and their corresponding responses.

Using PlantCARE to analyze the various *cis*-elements in the promoter sequence (upstream of 2,000 bp) of the *BnaPDX* gene, the results can be divided into five types of plant hormone response *cis*-elements (IAA, ABA, MeJA, GA, and SA), six types of environmental stress response *cis*-elements (Stress, Anaerobic, Circadian, Light, Drought, and Low-T), one specific transcription factor binding site (MYBHv1), and several gene-specific response *cis*-elements (**Figure 4**).

The results revealed that within the *BnaPDX* gene family of *B. napus*, there are 111 *cis*-elements linked to plant hormone response, 239 *cis*-elements associated with environmental stress response, and five *cis*-elements related to metabolism. Specifically, among these, 44 ABA response *cis*-elements represent the highest proportion within plant hormone response, whereas 169 light response *cis*-elements are the most prevalent in environmental stress response.

### 4.2 *BnaPDX1.3* of *B. napus* responds to waterlogging stress

To further analyze the response of *PDX* members to waterlogging stress, this study utilized RNA-seq data from *B. napus* G218 subjected to waterlogging treatment for 6 days, previously conducted in the field waterlogging experiment by our research group (Hong et al., 2023). We analyzed the expression of the *BnaPDX* gene family in *B. napus* under waterlogging stress and performed qRT-PCR verification of the *BnaPDX1.3* gene (*BnaAnng00210D*, *BnaA03g00380D*, *BnaC03g00280D*).

**Figure 5:**
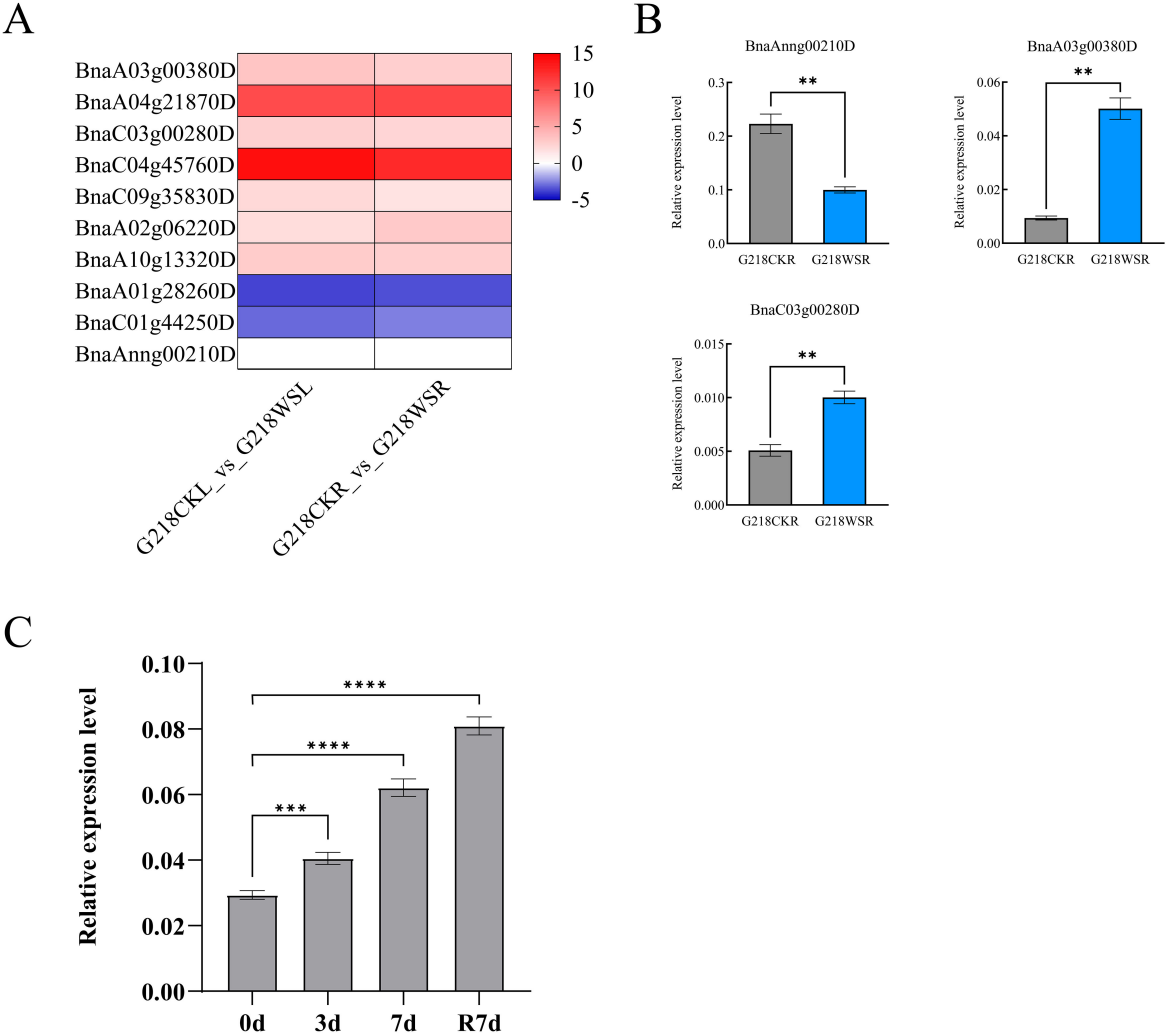
The *BnaPDX1.3* gene in *B. napus* is involved in the response to waterlogging stress. **(A)** RNA-seq analysis of *PDX* genes in *B. napus* G218 leaves and roots after 6 days of waterlogging stress. G218CKL denotes the control leaf, G218WSL the leaf after waterlogging stress, G218CKR the control root, and G218WSR the root after waterlogging stress. **(B)** qRT-PCR analysis of the *BnaPDX1.3* gene expression in *B. napus* G218 leaves after 6 days of waterlogging stress. The results of the qRT-PCR analysis are consistent with the RNA-seq. **(C)** qRT-PCR analysis of *BnaPDX1.3* gene expression in *B. napus* under waterlogging stress. qRT-PCR analysis of the *BnaPDX1.3* gene in *B. napus* at 0, 3, and 7 days of waterlogging stress, as well as after 7 days of reoxygenation following the end of stress. *p<0.05", "**p<0.01", ***p<0.001", ****p<0.0001".

Among the *BnaPDX* family genes, except for *BnaPDX1.2* (*BnaA01g28260D* and *BnaC01g44250D*) whose expression level was downregulated after 6 days of waterlogging stress, all other *BnaPDX* family genes showed upregulation, with *BnaPDX1.1* (*BnaA04g21870D* and *BnaC04g45760D*) exhibiting the most significant upregulation (**Figure 5A**). The qRT-PCR verification results were generally consistent with the RNA-seq data. Except for the *BnaAnng00210D* gene, whose expression decreased relatively after 6 days of waterlogging stress, the expression of the *BnaPDX1.3* gene was upregulated after stress in all other cases.

Interestingly, *BnaAnng00210D* exhibits the highest relative expression level, being 24 times higher than that of *BnaA03g00380D*. Its elevated expression level might account for its slightly downregulated expression under waterlogging stress (**Figure 5B**). Further investigation into the expression pattern of *BnaPDX1.3* under waterlogging stress revealed that the relative expression of *BnaPDX1.3* in G218 seedlings significantly increased after waterlogging treatment and continued to rise even 7 days after reoxygenation (**Figure 5C**), indicating that *BnaPDX1.3* in *B. napus* responds positively to waterlogging stress.

### 4.3 *BnaPDX1.3* enhances waterlogging tolerance in *B. napus*

**Figure 6:**
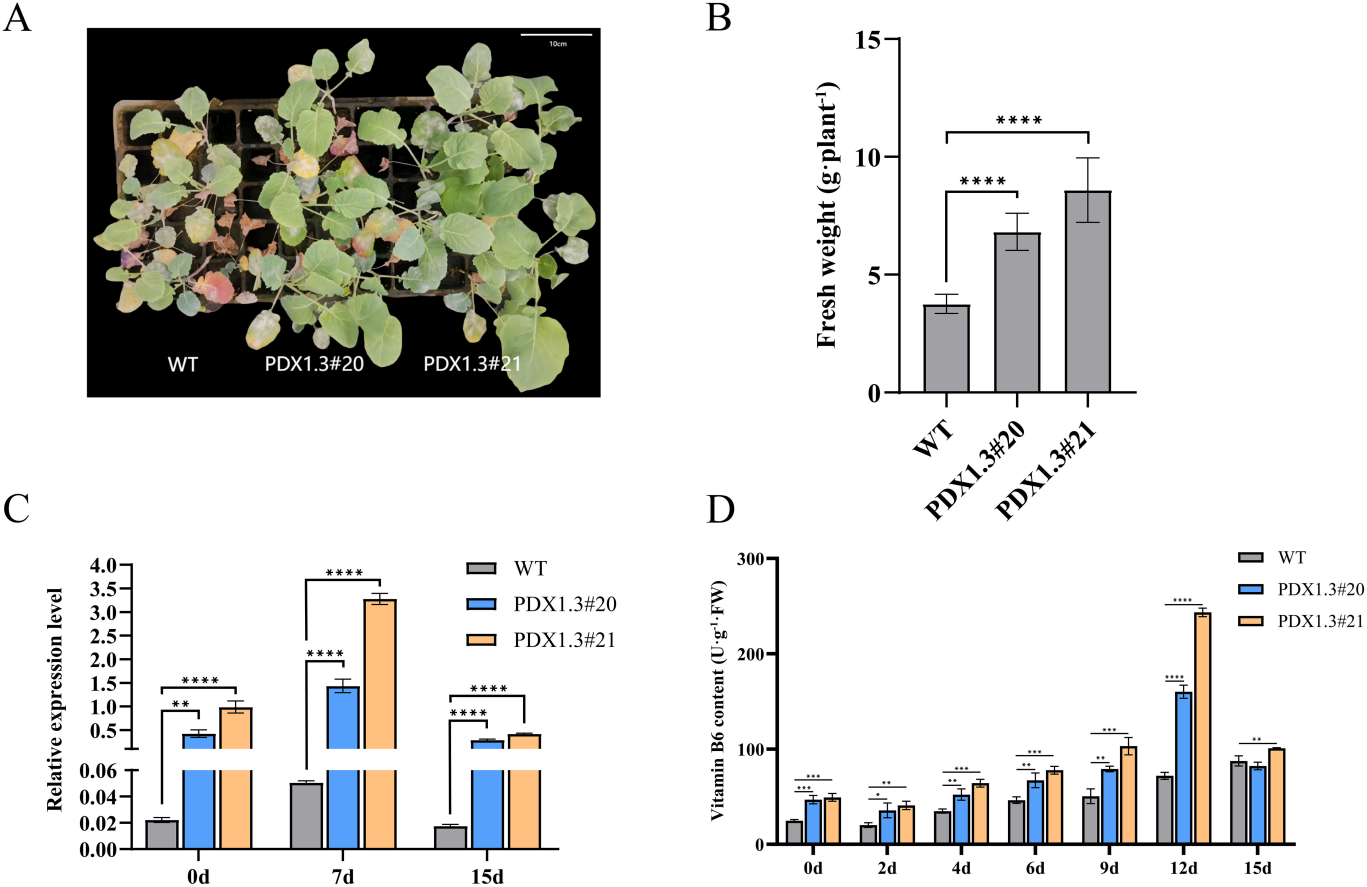
The *BnaPDX1.3* gene enhances waterlogging tolerance in *B. napus.* **(A)** Photographs of the *BnaPDX1.3* gene overexpressing *B. napus* and wild-type (WT) plants after 15 days of waterlogging stress. The photographs show that, compared to the overexpressing plants (PDX1.3#20 and PDX1.3#21), the wild-type plants exhibit more pronounced symptoms, including leaf shedding, purple-red discoloration, and yellowing. **(B)** Biomass analysis of the *BnaPDX1.3* gene overexpressing *B. napus* and wild-type plants after 15 days of waterlogging stress. The biomass of overexpressing plants was significantly higher than that of wild-type plants following waterlogging stress. **(C)** Quantitative expression analysis of the *BnaPDX1.3* gene in overexpressing and wild-type *B. napus* leaves under waterlogging stress. Overexpressing plants exhibited significantly higher expression levels of the *BnaPDX1.3* gene compared to wild-type plants. **(D)** Vitamin B6 (VB6) content in overexpressing and wild-type *B. napus* leaves under waterlogging stress. Plants with stronger *BnaPDX1.3* gene expression had higher VB6 content. This, along with their phenotype and biomass, corresponds to greater waterlogging tolerance. *p<0.05", "**p<0.01", ***p<0.001", ****p<0.0001".

To investigate whether overexpression of the *BnaPDX1.3* gene in *B. napus* plays a positive regulatory role in response to waterlogging stress, two groups of *BnaPDX1.3*-overexpressing *B. napus* (PDX1.3#20 and PDX1.3#21) and WT *B. napus* at the three-leaf and one-heart stage were subjected to 15 days of waterlogging stress (**Supplementary Figure S6**). Under waterlogging stress, the wild-type leaves exhibited significant leaf abscission, turning purplish-red and yellowing. In contrast, the leaves of *BnaPDX1.3*-overexpressing rapeseed PDX1.3#20 showed less abscission, purplish-red coloration, and yellowing, while the leaves of *BnaPDX1.3*-overexpressing *B. napus* PDX1.3#21 did not exhibit obvious waterlogging phenotypes (**Figure 6A**). In terms of biomass, the overexpressing plants exhibited significantly higher values after waterlogging compared to the wild type (**Figure 6B**).

qRT-PCR analysis showed that the expression level of the *BnaPDX1.3* gene in PDX1.3#20 overexpressing plants was 19 times higher than that in the wild type, while in PDX1.3#21, it was 45 times higher. After 7 days of waterlogging stress, the expression levels of the *BnaPDX1.3* gene in both overexpressing and wild-type plants were significantly upregulated. PDX1.3#20 was upregulated by 3.4 times, and PDX1.3#21 by 3.2 times, while the wild type showed only a 2.2-fold upregulation. After 15 days of waterlogging stress, the expression levels of the *BnaPDX1.3* gene in all three groups were downregulated to levels lower than those before treatment. However, the transgenic plants still maintained relatively high expression levels (**Figure 6C**).

Under waterlogging stress, the vitamin B6 content in the overexpressing plants was significantly higher than that in the wild-type plants, peaking after 12 days of stress. The VB6 peak in PDX1.3#21 was 3.4 times higher than in the wild type, while the VB6 peak in PDX1.3#20 was 2.2 times higher (**Figure 6D**). In summary, plants with higher expression of the *BnaPDX1.3* gene synthesize more vitamin B6 and exhibit greater tolerance to waterlogging.

### 4.4 Increased expression of *BnaPDX1.3* strengthens the plant antioxidant system

**Figure 7:**
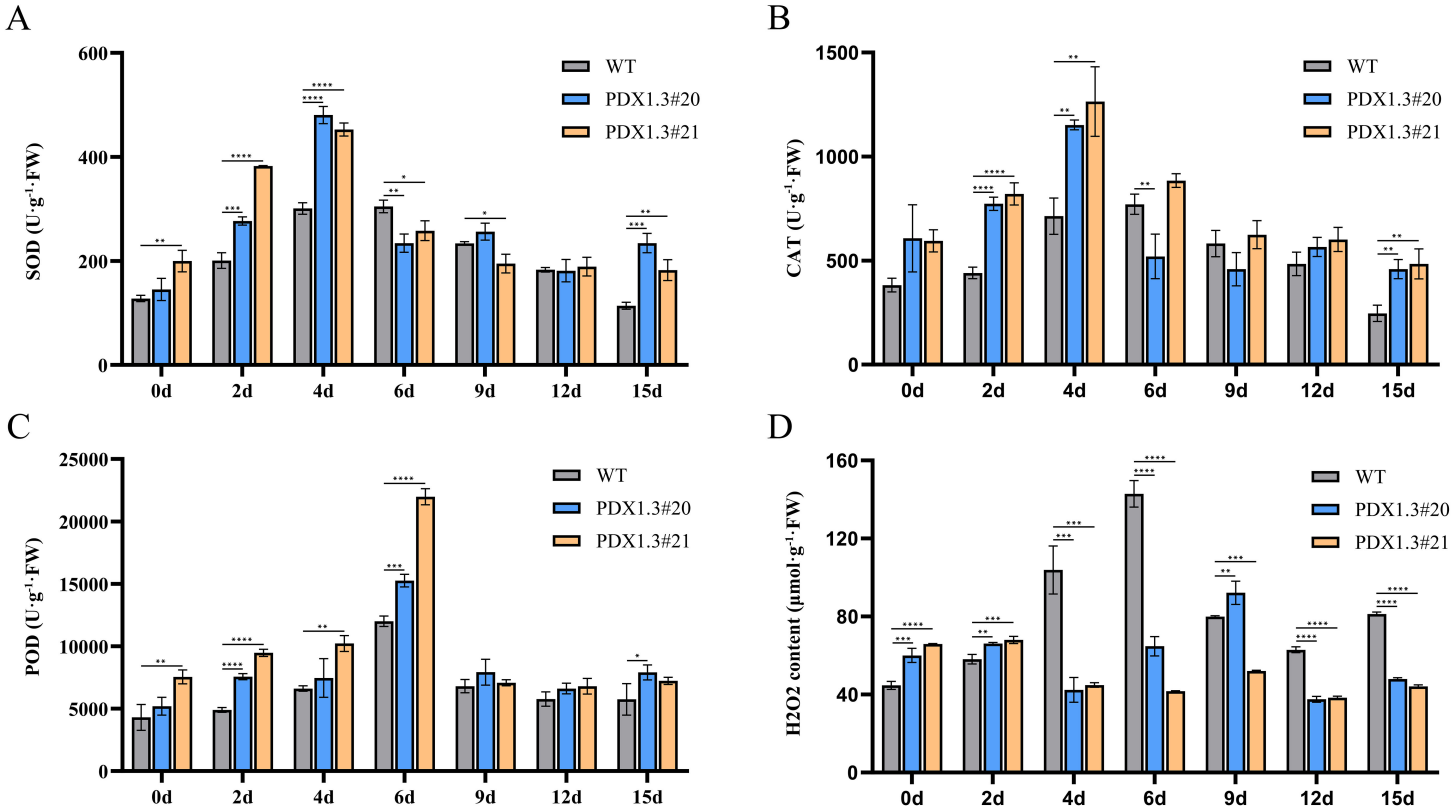
*BnaPDX1.3* gene enhances antioxidant enzyme activity in *B. napus*. **(A)** SOD activity under waterlogging stress in *BnaPDX1.3-*overexpressing *B. napus* and wild-type plants. SOD activity in overexpressing plants shows a significant difference compared to wild-type plants under waterlogging stress. **(B)** CAT activity in *BnaPDX1.3*-overexpressing *B. napus* and wild-type plants under waterlogging stress. CAT activity in overexpressing plants is generally significantly higher than in wild-type plants under waterlogging stress. **(C)** POD activity in *BnaPDX1.3*-overexpressing *B. napus* and wild-type plants under waterlogging stress. Overexpressing plants exhibit stronger POD activity compared to wild-type plants under waterlogging stress. **(D)** H_2_O_2_ content in *BnaPDX1.3-*overexpressing *B. napus* and wild-type plants under waterlogging stress. H_2_O_2_ content in overexpressing plants is significantly lower and more stable compared to wild-type plants under waterlogging stress. *p<0.05", "**p<0.01", ***p<0.001", ****p<0.0001".

Upon detecting antioxidant enzyme activity in plant leaves under waterlogging stress, it was found that transgenic plants exhibited stronger antioxidant enzyme activity and more stable H_2_O_2_ content. During the early stages of waterlogging (2–4 days), the SOD activity in transgenic plants was significantly higher than that in wild-type plants. It gradually returned to levels similar to those in wild-type plants between 6 and 12 days, but remained significantly higher at 15 days, maintaining a high level of SOD activity (**Figure 7A**).

During the early stages of waterlogging (2–4 days), the CAT activity in transgenic plants was significantly higher than that in wild-type plants. It gradually decreased, but the trend was more stable in transgenic plants. At 15 days, CAT activity remained significantly higher in transgenic plants, maintaining a high level of CAT activity (**Figure 7B**).

From 2 to 6 days, the POD activity in transgenic plants was significantly higher than that in wild-type plants. During this period, all three groups of plants showed a gradual increase, reaching the same level at 9 days, after which the activity stabilized (**Figure 7C**).

After 4 days of waterlogging stress, the H_2_O_2_ content in transgenic plants rapidly decreased, while in wild-type plants, it rapidly increased, with their H_2_O_2_ content significantly higher than that of transgenic plants. At 6 days, the H_2_O_2_ content of wild-type plants reached its peak and remained elevated throughout the later stages of stress, while the H_2_O_2_ content in *BnaPDX1.3*-overexpressing plants had stabilized after 4 days and remained significantly lower than in wild-type plants, maintaining a low level throughout the later stages of stress (**Figure 7D**).

In summary, high-level expression of *BnaPDX1.3* helps plants establish a stronger and more efficient antioxidant system under waterlogging stress, effectively preventing the accumulation of ROS and subsequent oxidative damage.

## 5 Discussion

In China, *B. napus* is the second-largest oilseed crop, with a planting area of approximately 6.57 million hectares, accounting for 38.6% of the country's total oil crop production (Wang et al., 2022; Li and Wang, 2022). The Yangtze River Basin is the main producing area for *B.napus* in China, where 91% of the total oilseed rape in China is produced (Tao et al., 2015), *B. napus* production in the region primarily follows a rice–rapeseed rotation. The soil has a relatively high water-holding capacity, and the rapeseed growing period coincides with the rainy season in the basin, which is characterized by frequent high-intensity rainfall, making the region prone to waterlogging (Tao et al., 2024; Zhu et al., 2019). Waterlogging can affect root vitality and growth, photosynthetic efficiency, and the physiological metabolism of rapeseed, ultimately affecting yield and quality (Xu et al., 2018; Ploschuk et al., 2018).

Vitamin B6, as an endogenous growth regulator, is considered an antioxidant in plants, influencing physiological metabolism, growth, development, and stress resistance (Colinas et al., 2016; Moccand et al., 2014; Zhang et al., 2014; Huang et al., 2013). The *PDX* family genes, key players in the vitamin B6 synthesis pathway, have been shown to be involved in responding to various environmental stresses in plants. Studies have shown that the three *PDX1* and *PDX2* genes in *Arabidopsis thaliana* respond to strong light, cold, drought, and ozone stress (Bagri et al., 2018; Leasure et al., 2011). The *pdx1.3* mutant of *Arabidopsis thaliana* is more sensitive to salt, oxidative, and osmotic stress (Titiz et al., 2006; Moccand et al., 2014). Currently, research on plant *PDX* genes has primarily focused on *Arabidopsis thaliana*, and no studies have reported on the *PDX* genes in *B. napus*. As a gene involved in vitamin B6 synthesis, the effect of *BnaPDX1.3* on *B. napus’s* response to waterlogging stress has not been fully elucidated. Therefore, this study aims to explore the role of the *BnaPDX1.3* gene in *B. napus’s* response to waterlogging stress by analyzing the phenotype, as well as physiological and biochemical indicators, of T1 generation *BnaPDX1.3*-overexpressing rapeseed plants under waterlogging stress.

The research findings indicate that, after a 15-day waterlogging stress experiment, the transgenic rapeseed plants with higher expression levels of the *BnaPDX1.3* gene exhibited healthier leaves and greater biomass compared to wild-type plants, suggesting that the *BnaPDX1.3* gene enhances the waterlogging tolerance of *B. napus*. To delve deeper into the functional mechanism of the *BnaPDX1.3* gene in *B. napus*, this study examined the changes in VB6 content under waterlogging stress. The results revealed that the VB6 content in the transgenic plants was generally higher than that in the wild-type plants, with the peak VB6 content in the transgenic plants on day 12 being 3.4 times higher than that in the wild-type plants.

H_2_O_2_, as a plant stress signaling molecule and reactive oxygen species, can reflect the plant’s ability to respond to and resist stress. In this study, the analysis of H_2_O_2_ content after waterlogging showed that the wild-type plants accumulated excessive H_2_O_2_ 6 days after waterlogging and maintained high levels of H_2_O_2_ throughout the later stages of the stress, unable to effectively prevent cellular oxidative damage. However, the *BnaPDX1.3*-overexpressing plants maintained lower H_2_O_2_ levels under waterlogging conditions. The analysis of antioxidant enzyme activity indicated that the *BnaPDX1.3*-overexpressing plants had significantly higher SOD, POD, and CAT enzyme activities under waterlogging stress compared to the wild-type control plants, demonstrating a stronger antioxidant capacity. This effectively eliminates ROS generated by waterlogging, reduces oxidative damage, and thereby enhances plant tolerance to waterlogging.

**Figure 8:**
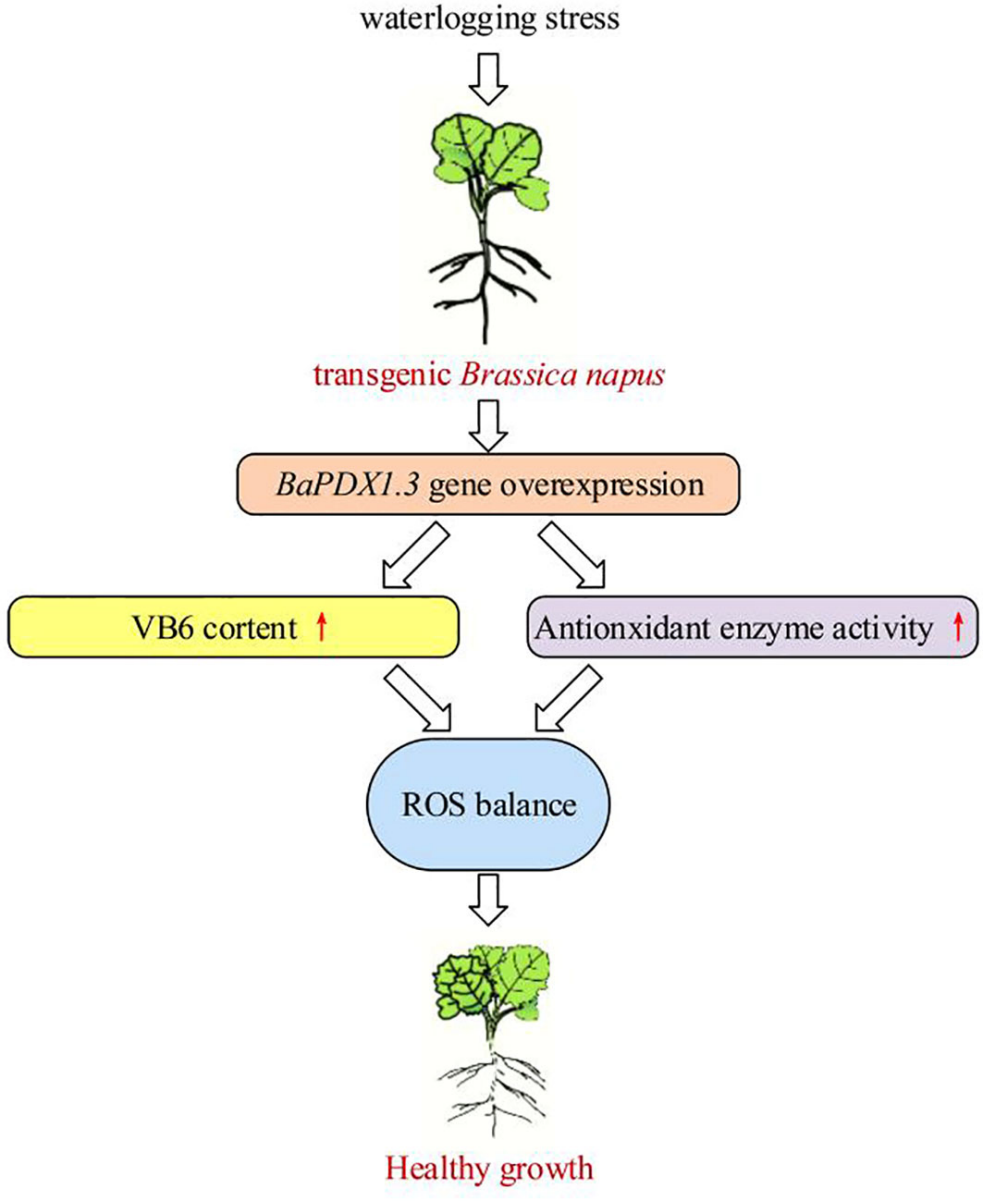
Impact of overexpression of *BnaPDX1.3* in *B. napus* on plant waterlogging tolerance. *BnaPDX1.3*-overexpressing *B. napus* plants exhibit high expression levels of the *BnaPDX1.3* gene under waterlogging stress, synthesize high levels of VB6, and significantly enhance antioxidant enzyme activity. This results in a stable dynamic balance of ROS under waterlogging stress, supporting healthy growth.

We conclude that overexpressing plants, with high *BnaPDX1.3* gene expression, synthesize more VB6, exhibit stronger antioxidant enzyme activity, and possess a more efficient and stable ROS scavenging system, thereby demonstrating healthy growth under waterlogging stress. In summary, the *BnaPDX1.3* gene enhances the waterlogging tolerance of *B. napus*, which is of great significance for its response to waterlogging stress. The event model revealed in this study is shown in **Figure 8**. Additional studies have shown that VB6 can act as an antioxidant, participating in the scavenging of ROS accumulation (Lu et al., 2022; Danon et al., 2005; Zhang et al., 2022). Moreover, *pdx1* mutants impair IAA biosynthesis (Boycheva et al., 2015; Chen and Xiong, 2009), and impaired VB6 biosynthesis can inhibit ABA biosynthesis (Liu et al., 2022). In summary, VB6 participates in plant responses to adverse stress by regulating plant hormone synthesis and antioxidant systems, which may explain why overexpressing *BnaPDX1.3* plants exhibit stronger waterlogging tolerance.

## 6 Conclusion

The bioinformatics analysis of the *PDX* gene family and the waterlogging stress experiment analysis with the G218 wild type in this study indicate that the *BnaPDX1.3* gene is involved in the response of *B. napus* to waterlogging stress. To further investigate the regulatory role of the *BnaPDX1.3* gene in waterlogging stress, this study generated *BnaPDX1.3*-overexpressing *B. napus* plants through *Agrobacterium*-mediated transformation. After positive detection and screening, T1 generation positive seedlings were obtained. The T1 generation *BnaPDX1.3*-overexpressing *B. napus* plants were subjected to a 15-day waterlogging stress treatment. It was observed that *BnaPDX1.3*-overexpressing *B. napus* plants had healthier leaves and greater biomass compared to wild-type plants, exhibiting better waterlogging tolerance. Additionally, they displayed a stronger antioxidant system and a more stable ROS content under waterlogging stress. The results of this study indicate that the *BnaPDX1.3* gene can enhance the waterlogging tolerance of *B. napus*, which is of great significance for its response to waterlogging stress.

## Data Availability

The original contributions presented in the study are publicly available. This data can be found here: https://www.ncbi.nlm.nih.gov/, accession number PRJNA898876.
