# Supplementary material for: Genome-wide identification of PDX and expression analysis under waterlogging stress exhibit stronger waterlogging tolerance in transgenic Brassica napus plants overexpressing the BnaPDX1.3 gene compared to wild-type plants
Source: Front Plant Sci. 2025 Feb 12;16:1533219. doi: 10.3389/fpls.2025.1533219 (PMC11863972; doi:10.3389/fpls.2025.1533219)
Supplement: Supplementary file 1 [file Table1.docx]

**Table S1** The primer sequences used for qRT-PCR analysis.

| Primer name | Primer sequences (5' to 3') |
| --- | --- |
| BnaAnng00210D-F | CGTAGTGGCTGTGTACGGT |
| BnaAnng00210D-R | ACGTCCATGATAACTCCGCC |
| BnaA03g00380D-F | ATCAGGACGAAAGGTGAGGC |
| BnaA03g00380D-R | GAGATCGTAAGGAGCGGCAA |
| BnaC03g00280D-F | TCATGGCTTTGGAGCGAGTCC |
| BnaC03g00280D-R | TTCGTCGGCGAGAGTCAGGA |
| BnACTIN-F | GGTTGGGATGGACCAGAAGG |
| BnACTIN-R | TCAGGAGCAATACGGAGC |

**Table S2** The primer sequences used for amplifying a target fragment with restriction sites.

| Primer name | Primer sequences (5' to 3') |
| --- | --- |
| PDX1.3-GF | GGGGTACCCCATGGAAGGAACAAGCGTCGTGG |
| PDX1.3-GR | GCTCTAGAGCTCACTCGGAGCGGTTAGCAAAC |

**Table S3** The primer sequences used for detecting positive transgenic plants.

| Primer name | Primer sequences (5' to 3') |
| --- | --- |
| NPTII-F | ACTGGGCACAACAGACAATCG |
| NPTII-R | GCATCAGCCATGATGGATACTTT |
| PDX1.3-T1F | CGAACTGAACGACGGGAAGA |
| PDX1.3-T1R | TCTCGAGCTTTCGCGAGCTC |
