# Supplementary material for: Genome-wide identification of PDX and expression analysis under waterlogging stress exhibit stronger waterlogging tolerance in transgenic Brassica napus plants overexpressing the BnaPDX1.3 gene compared to wild-type plants
Source: Front Plant Sci. 2025 Feb 12;16:1533219. doi: 10.3389/fpls.2025.1533219 (PMC11863972; doi:10.3389/fpls.2025.1533219)
Supplement: Supplementary file 2 [file DataSheet1.pdf]

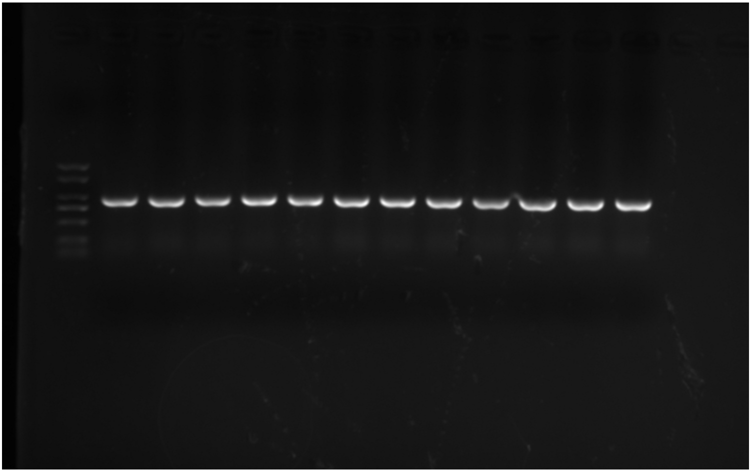

Figure S1. The target fragment with restriction sites

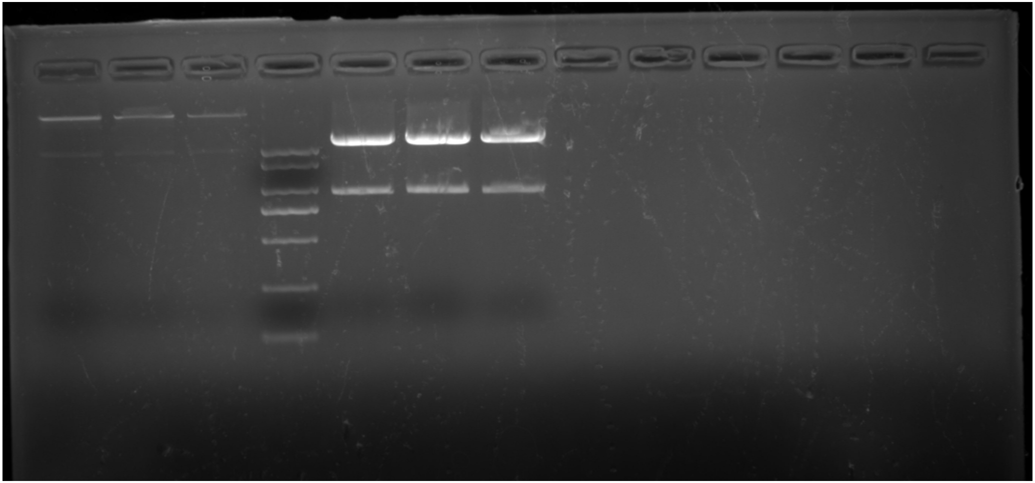

Figure S2. Double-enzyme digestion was used to validate the constructed overexpression vector (Three on the right).

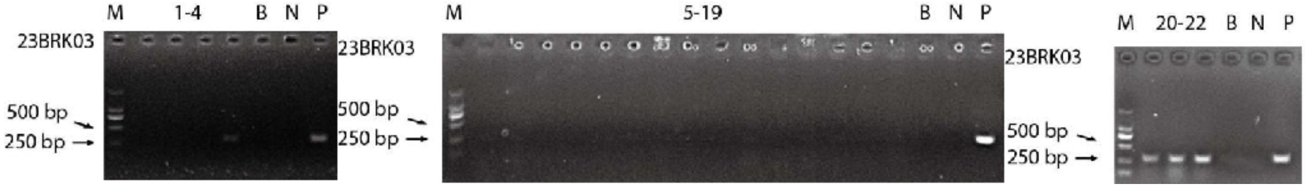

Figure S3. Positive detection of T0 transgenic seedlings.

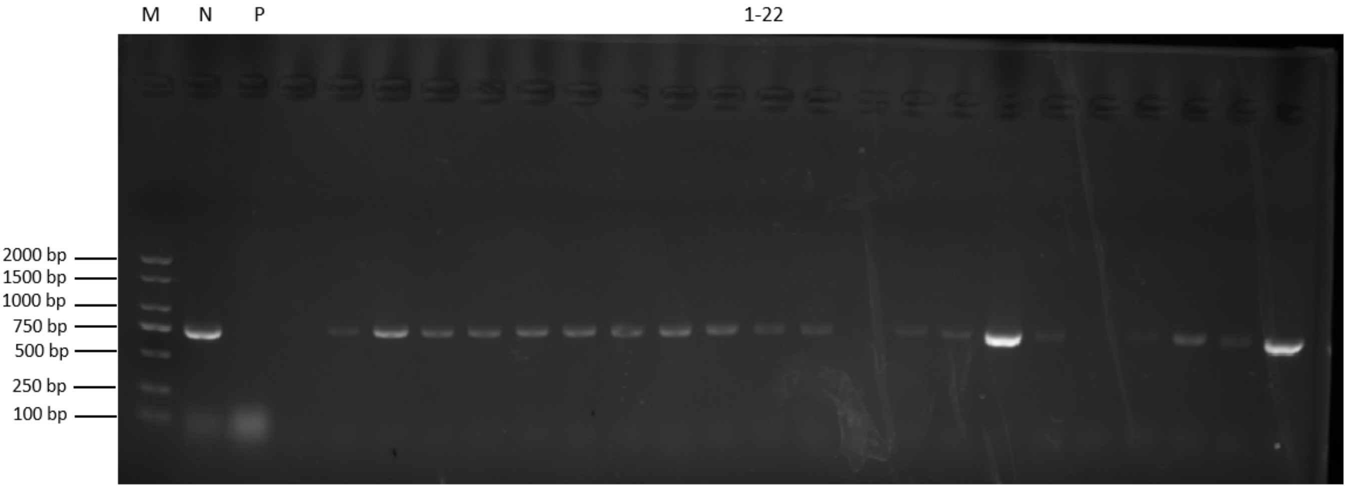

Figure S4. Positive detection of the T1 generation PDX1.3#20 transgenic plants.

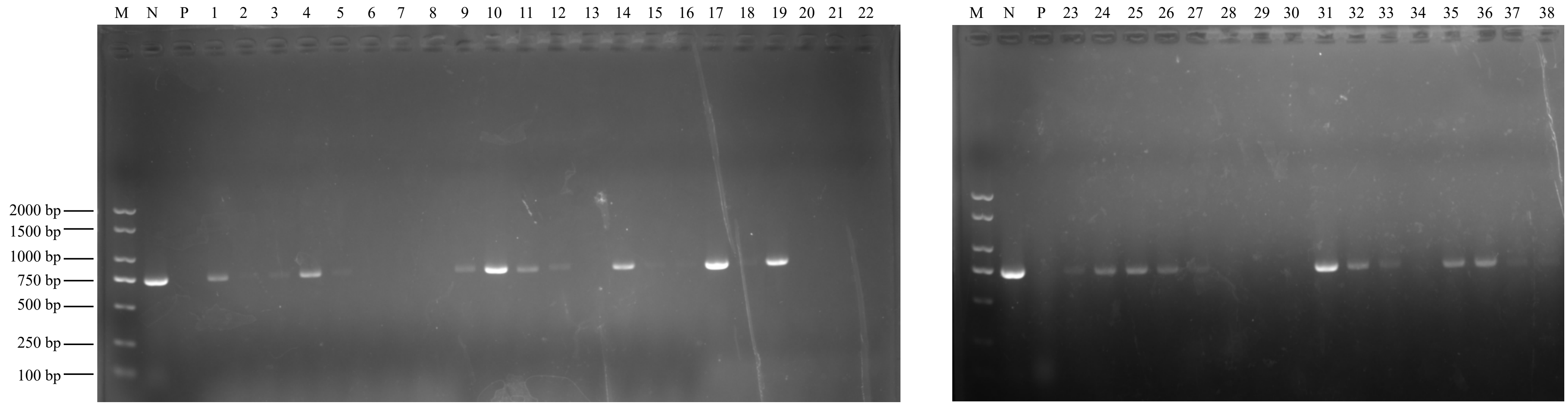

Figure S5. Positive detection of the T1 generation PDX1.3#21transgenic plants.

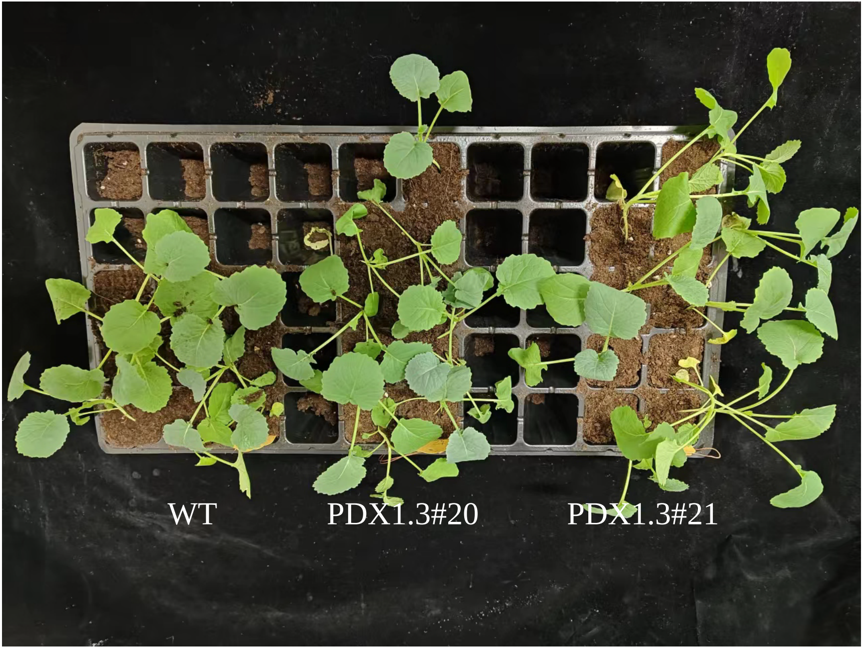

Figure S6. The phenotype of plants before waterlogging treatment.
